# Efficacy and safety of novel-targeted drugs in the treatment of pulmonary arterial hypertension: a Bayesian network meta-analysis

**DOI:** 10.1080/10717544.2021.1927243

**Published:** 2021-06-01

**Authors:** Wenhai Fu, Wenjun He, Yuexin Li, Yangxiao Chen, Jingyi Liang, Hui Lei, Lin Fu, Yanghang Chen, Ni Ren, Qian Jiang, Yi Shen, Ran Ma, Tao Wang, Xinni Wang, Nuofu Zhang, Dakai Xiao, Chunli Liu

**Affiliations:** a Department of Medicine, First Clinical School, Guangzhou Medical University, Guangzhou, China; b Department of Respiratory and Critical Care Medicine, The First Affiliated Hospital of Guangzhou Medical University, State Key Laboratory of Respiratory Disease, National Clinical Research Center for Respiratory Disease, Guangdong Key Laboratory of Vascular Disease, Guangzhou Institute of Respiratory Health, Guangzhou, China

**Keywords:** Pulmonary arterial hypertension, network meta-analysis, targeted drug, treatment

## Abstract

**Background:** Pulmonary arterial hypertension (PAH) is a severe and fatal clinical syndrome characterized by high blood pressure and vascular remodeling in the pulmonary arterioles, which is also a rapidly progressing disease of the lung vasculature with a poor prognosis. Although PAH medication made great advances in recent years, the efficacy and safety of the medication are unsatisfactory. Therefore, we aimed to update and expand previous studies to explore the efficacy and safety of PAH-targeted medications. **Methods:** Relevant articles were searched and selected from published or publicly available data in PubMed, Cochrane Library, CNKI, PsycInfo, and MEDLINE (from inception until October 1^st^, 2020). To assess the efficacy and safety of PAH therapies, five efficacy outcomes [6-minute walking distance (6MWD), mean pulmonary arterial pressure (mPAP), WHO functional class (WHO FC) improvement, clinical worsening, death] and two safety outcomes [adverse events (AEs), serious adverse events (SAEs)] were selected. And 6MWD was regarded as the primary efficacy outcome.

**Results:** 50 trials included with 10 996participants were selected. In terms of efficacy, all targeted drugs were more effective than placebo. For 6MWD, Bosentan + Sildenafil, Sildenafil, Bosentan + Iloprost were better than others. Bosentan + Iloprost and Bosentan + Sildenafil were better for mPAP. Bosentan + Iloprost and Ambrisentan + Tadalafil were more effective in improving WHO FC. Bosentan + Tadalafil and Bosentan + Iloprost had the Ambrisentan probability to reduce the incidence of clinical worsening. It is demonstrated that Ambrisentan had clear benefits in reducing all-cause mortality. In terms of safety, no therapies had been shown to reduce the incidence of SAEs significantly, and Ambrisentan + Tadalafil significantly increased the incidence of AEs.

**Conclusions:** Phosphodiesterase 5 inhibitor (PDE5i) + Endothelin Receptor Antagonists (ERA) seems to be better therapy for PAH. Prostacyclin analogs (ProsA) + ERA appear promising, though additional data is warranted.

**Registration** PROSPERO CRD42020218818.

## Introduction

Pulmonary arterial hypertension (PAH) is a lethal disease characterized by high pulmonary arterial pressure (Dodson et al., 2018), which is also a rapidly progressing disease of the lung vasculature with a poor prognosis (Prins and Thenappan, 2016). When left untreated, it may ultimately lead to right heart failure and death (Querejeta Roca et al., 2015). The therapeutic methods of PAH are intricate, including PAH risk assessment, acute pulmonary vascular reaction assessment, general treatment, supportive therapy, targeted drug therapy, interventional and surgical treatment, and efficacy evaluation (Kusunose et al., 2019). With the development of the research in pathogenesis and pathophysiology of PAH, there has recently been substantial progress in developing new therapeutic strategies for the management of patients with PAH (Montani et al., 2014). The approved targeted drugs including Endothelin Receptor Antagonists (ERA) (Bosentan, Ambrisentan, Macitentan), Phosphodiesterase 5 inhibitor (PDE5i) (Sildenafil, Tadalafil, Vardenafil), soluble guanylate cyclase (SGC) (Riociguat), Prostacyclin analogue (ProsA) (Epoprostenol, Iloprost, Treprostinil, Beraprost, Selexipag) (O'Connell et al., 2013). These targeted drugs have been shown to have the ability of relieving PAH symptoms and further improving exercise capacity and hemodynamics (Lajoie et al., 2016; Zheng et al., 2018). Although significant PAH treatment advances have been made in recent years, the effectiveness and safety are unsatisfactory. Therefore, clinicians need sufficient evidence to make the optimal choice for each PAH patient. Thus, to provide a more convincing guideline for the clinical practice of PAH, our goal is to perform a network meta-analysis systematic review combining direct and indirect evidence to explore targeted drugs’ efficacy and safety.

## Methods

The systematic review protocol has been registered with the International Prospective Register of Systematic Reviews Database (PROSPERO). The registration number is CRD42020218818.

## Search strategy

We searched the database including PubMed, Cochrane Library, CNKI, PsycInfo, and MEDLINE (from inception until October 1, 2020). We used the following terms together with their corresponding synonyms in our searches: (‘pulmonary arterial hypertension’ OR ‘PAH’) AND (‘Bosentan’ OR ‘Ambrisentan’ OR ‘Macitentan’ OR ‘Sildenafil’ OR ‘Tadalafil’ OR ‘Vardenafil’ OR ‘Riociguat’ OR ‘Epoprostenol’ OR ‘Iloprost’ OR ‘Treprostinil’ OR ‘Beraprost’ OR ‘Selexipag’). We screened the reference list for each relevant article one by one to check their qualifications. Then, two investigators retrieved the relevant articles independently. If there had been different opinions, we settled them through discussion. Additionally, the literature review searches will be updated at the end of the process.

## Selection criteria

Studies will be included if they were conforming to the following criteria: (1) randomized controlled trials (RCTs) as study design and comparator was either a PAH-targeted drug or placebo; (2) patients were diagnosed as group 1 PH according to the clinical classification of PAH; (3) patients with PAH (group 1 PH) were primarily adults (allowing patients under 14 years old if most of the participants were adults); (4) the minimum follow-up period was 12 weeks.

The exclusion criteria were: (1) non-RCTs, studies with insufficient data, duplicated publications, conference reports, systematic reviews; (2) trials restricted neonatal or pediatric patients; (3) studies on patients from the remaining WHO PH groups or no data for PAH were available.

## Data extraction

Two investigators independently reviewed all relevant articles to perform the data retrieval and eligibility assessment with standardized data abstraction forms. Any disagreement was resolved after mutual agreement and discussion or with the help of the third investigator.

## Outcome measures

The effective primary outcome of this network meta-analysis was 6MWD. The effective secondary outcomes included mPAP, WHO functional class (WHO FC) improvement, clinical worsening, and all-cause death. The safety outcomes were AEs and SAEs.

In terms of continuous outcome measures (i.e. 6MWD, mPAP), differences in the mean change from baseline (mean change) separately for the treatment and comparator arm and their standard deviation (SD) were extracted. When the mean and SD are not available, all data in the manuscript are reported in the forms of median [range, size of a sample or the sample], median [interquartile range] and 95% CIs (P value) (Hozo et al., 2005; Wan et al., 2014). For dichotomous outcomes (i.e. death, clinical worsening, WHO FC improvement, AEs, SAEs), the total number of patients (N) and the number of patients with events (r) were extracted or calculated for each treatment.

## Quality assessment

The risk of bias for individual studies was assessed according to the Cochrane Handbook method for Systematic Reviews of Interventions. The following domains were evaluated: random sequence generation (selections bias), allocation concealment (selections bias), blinding of participants and personnel (performance bias), blinding of outcome assessment (detection bias), incomplete outcome data (attrition bias), selective reporting (reporting bias), and other bias. The overall risk of bias will be determined as low (all items were low risk, or at least five items were low risk and the remaining two unclear), unclear (>2 items were unclear risk), and high (≥1 quality dimension suggested high bias) (Wei et al., 2016).

## Network meta-analysis

A network evidence diagram was constructed by using STATA16 software. A Bayesian network meta-analysis was used to compare the differences between different interventions. Results were presented as the pooled estimates of odds ratios (ORs) or weighted mean difference (WMD) (95% CI). The node splitting method was used to determine the consistency between direct or indirect evidence (Zhang and Xiao, 2018). Based on the results, to choose a consistent or inconsistent model, a consistency model was selected for further analysis when the results of node-splitting are *P* > 0.05 (Wang et al., 2018). To help interpret ORs or WMDs, the surface under the cumulative ranking curves (SUCRA) (Salanti et al., 2011; Mbuagbaw et al., 2017) was used to calculate each intervention’s probability. The SUCRA value ranges from 1 to 0. The larger the SUCRA value, the better the rank of the intervention. In contrast, the SUCRA was plotted for ranking, where a lower SUCRA value indicated a higher risk of adverse events (AEs). However, the interpretation of SUCRA needs to be prudently interpreted based on a statistical difference. We evaluated whether treatment effects for 6MWD were robust in subgroup analyses by using a method of administration. Subgroup analyses were conducted by using the Bayesian Markov Chain Monte Carlo method in OpenBUGS. The sensitivity assessment of our conclusions was restricted by the multicenter.

## Results

**Figure 1.**
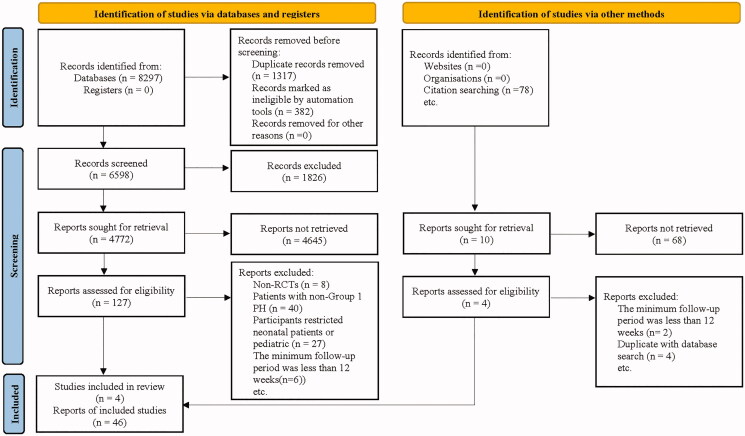
Flowchart for the process of screening out the included studies.

**Figure 2.**
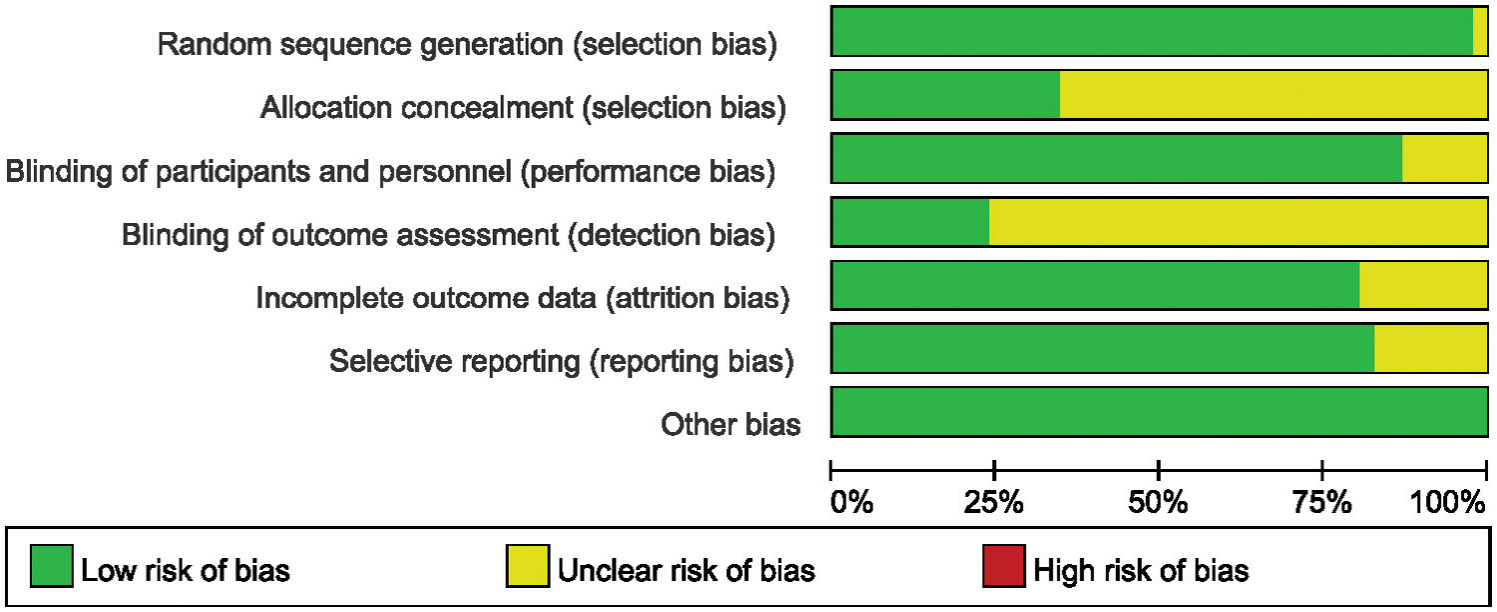
Risk of bias graph: review authors’ judgments about each risk of bias item presented as percentages across all included studies.

The search identified 8375 articles, and 137 potentially qualified articles were retrieved. Finally, a total of 50 articles with 10 996 participants were included in the study. The selection process details were shown in Figure 1. The primary characteristics of these studies were shown in Table 1. Patient characteristics of subjects included in the selected RCTs were shown in Table 2. Changes in 6MWD were shown in Supplementary Table 1. Figure 2 demonstrated an assessment of bias risk by the Cochrane risk of bias tool.

**Table 1.** The main characteristics of the included randomized controlled trials.

First author (year)	Study design	Aetiology	Outcome assessment time	Treatment		Comparator	
				Intervention	*n*	Intervention	*n*
McLaughlin et al. (2010)	MC, DB	IPAH, APAH	12 weeks	Treprostinil (18–54 ug)	115	Placebo	120
White et al. (2019)	MC, DB	IPAH, APAH	24 weeks	Treprostinil (0.125 mg)	346	Placebo	344
Jing et al. (2013)	MC, DB	IPAH, APAH	12 weeks	Treprostinil (1 -–12 mg)	233	Placebo	116
Hiremath et al. (2010)	MC, DB	IPAH, APAH	12 weeks	Treprostinil (4–100 ng/kg/min)	30	Placebo	14
Simonneau et al. (2012)	MC, DB	IPAH, APAH, HPAH	17 weeks	Selexipag (400–800 ug)	33	Placebo	10
Coghlan et al. (2018)	MC, DB	IPAH, APAH, HPAH, Drugs and toxins induced	12 weeks	Selexipag (200–1600 ug)	179	Placebo	197
Sitbon et al. (2015)	MC, DB	IPAH, APAH, HPAH, Drugs and toxins induced	26 weeks	Selexipag (200–1600 ug)	574	Placebo	582
Galie et al. (2009)	MC, DB	IPAH, APAH	12 weeks	Tadalafil (20 mg)	82	Placebo	82
Bermejo et al. (2018)	MC, DB	APAH	24 weeks	Sildenafil (100 mg)	104	Placebo	96
Galiè et al. (2015)	MC, DB	IPAH, APAH	12 weeks	Riociguat (2.5 mg)	12	Placebo	6
Rubin et al. (2015)	MC, DB	IPAH, APAH, HPAH	12 weeks	Riociguat (2.5 mg)	231	Placebo	109
Galiè et al. (2008)	MC, DB	IPAH, APAH, Drugs and toxins induced	12 weeks	Ambrisentan (5 mg)	67	Placebo	130
Galiè et al. (2008)	MC, DB	IPAH, APAH, Drugs and toxins induced	12 weeks	Ambrisentan (5 mg)	63	Placebo	65
White et al. (2019)	MC, DB	IPAH, APAH	24 weeks	Ambrisentan (10 mg) + Tadalafil (40 mg)	253	Placebo Ambrisentan (10 mg) Tadalafil (40 mg)	247 126 121
Galiè et al. (2015)	MC, DB	IPAH, APAH, HPAH, Drugs and toxins induced	16 weeks	Ambrisentan (10 mg) + Tadalafil (40 mg)	253	Placebo Ambrisentan (10 mg) Tadalafil (40 mg)	247 126 121
Kuwana et al. (2020)	MC, DB	HPAH	16 weeks	Ambrisentan (10 mg) + Tadalafil (40 mg)	117	Ambrisentan (10 mg) Tadalafil (40 mg)	52 47
Baughman et al. (2014)	MC, DB	HPAH	16 weeks	Bosentan (62.5 mg up to 125 mg)	23	Placebo	12
Rubin et al. (2002)	MC, DB	APAH	12 weeks	Bosentan (125 mg)	74	Placebo	69
Galiè et al. (2002)	MC, DB	APAH	16 weeks	Bosentan (62.5–125 mg)	37	Placebo	17
Badesch et al. (2002)	MC, DB	IPAH, APAH	12 weeks	Bosentan (62.5–125 mg)	21	Placebo	11
Galiè et al. (2008)	MC, DB	IPAH, APAH	32 weeks	Bosentan (62.5–125 mg)	93	Placebo	92
Ni and Wa (2018)	SC, NR	APAH	24 weeks	Bosentan (62.5–125 mg)	26	Placebo	26
McLaughlin et al. (2015)	MC, DB	IPAH, APAH, HPAH, Drugs and toxins induced	32 weeks	Sildenafil (20 mg) + Bosentan (125 mg)	159	Sildenafil (20 mg)	175
Iversen et al. (2010)	SC, DB	APAH	12 weeks	Sildenafil (25 mg up to 50 mg) + Bosentan (62.5 mg up to 125 mg)	10	Bosentan (62.5 mg up to 125 mg) + Placebo	10
Vizza et al. (2017)	MC, DB	IPAH, HPAH	12 weeks	Sildenafil (20 mg) + Bosentan (62.5 mg or 125 mg)	50	Bosentan (62.5 mg or 125 mg) + Placebo	53
Ling-Yun et al. (2016)	SC, DB	APAH	Ambiguous	Sildenafil (20 mg) + Bosentan (125 mg)	31	Sildenafil (20 mg)	30
Pulido et al. (2013)	MC, DB	IPAH, APAH, HPAH, Drugs and toxins induced	24 weeks	Macitentan (3 mg and 10 mg)	492	Placebo	250
Sitbon et al. (2019)	SC, DB	APAH	12 weeks	Macitentan (10 mg)	43	Placebo	42
Jansa and Pulido (2018)	MC, DB	IPAH, APAH, HPAH, Drugs and toxins induced	24 weeks	Macitentan (10 mg)	242	Placebo	250
De-Zhen and An-Meng (2020)	SC, NR	IPAH, APAH	24 weeks	Tadalafil (10 mg up to 20 mg) + Bosentan (62.5 mg up to 125 mg)	40	Tadalafil (10 mg up to 20 mg)	40
Jian-zhou et al. (2018)	SC, NR	APAH	24 weeks	Tadalafil (10 mg) + Bosentan (62.5 mg up to 125 mg)	43	Bosentan (62.5 mg up to 125 mg)	43
Galie et al. (2002)	MC, DB	IPAH, APAH	12 weeks	Beraprost (80 ± 35ug)	65	Placebo	65
McLaughlin et al. (2006)	MC, DB	IPAH, HPAH	12 weeks	Iloprost (5ug) + Bosentan (125 mg)	34	Bosentan (125 mg)	33
Hoeper et al. (2006)	MC, DB	IPAH	12 weeks	Iloprost (5ug) + Bosentan (125 mg)	19	Bosentan (125 mg)	21
Badesch (2000)	MC, DB	APAH	12 weeks	Epoprostenol (5 ng/kg)	60	Placebo	55
Barst et al. (1996)	MC, NR	IPAH	12 weeks	Epoprostenol (2 ng/kg)	41	Placebo	40
Barst (1997)	MC, NR	IPAH	48 weeks	Epoprostenol (2 ng/kg)	11	Placebo	12
Cheitlin (2006)	MC, DB	IPAH, APAH	12 weeks	Sildenafil (40 mg)	67	Placebo	70
Wilkins et al. (2005)	SC, DB	IPAH, APAH	14 weeks	Bosentan (62.5 mg up to 125 mg)	12	Sildenafil (50 mg)	14
Ronald (2004)	MC, DB	APAH	12 weeks	Treprostinil (1.25 ng/kg-2.5 ng/kg up to 22.5 ng/kg)	41	Placebo	49
Tapson et al. (2012)	MC, DB	IPAH, APAH, HPAH	16 weeks	Treprostinil (0.5 mg-1 mg)	174	Placebo	176
Simonneau et al. (2014)	MC, DB	IPAH, HPAH, APAH, Drugs and toxins induced	16 weeks	Sildenafil (20 mg)	134	Placebo	131
Barst et al. (2003)	MC, DB	APAH	48 weeks	Beraprost (120ug)	60	Placebo	56
Humbert et al. (2004)	MC, DB	IPAH, APAH	16 weeks	Epoprostenol (2 ng/kg/min up to 14 ± 2 ng/kg/min) + Bosentan (62.5 mg up to 125 mg)	22	Epoprostenol (2 ng/kg/min up to 14 ± 2 ng/kg/min) + Placebo	11
Jian-Yong et al. (2018)	SC, NR	APAH	12 weeks	Sildenafil (25 mg)	41	Sildenafil (20 mg) + Bosentan (62.5 mg)	35
Tapson et al. (2013)	MC, DB	IPAH, APAH, HPAH	16 weeks	Treprostinil (3.1 ± 1.9 mg)	157	Placebo	153
Zhuang et al. (2014)	MC, DB	IPAH, APAH, HPAH	16 weeks	Ambrisentan (10 mg) + Tadalafil (40 mg)	60	Ambrisentan (10 mg) + Placebo	60
Jing et al. (2011)	MC, DB	IPAH, APAH	12 weeks	Vardenafil (5 mg)	44	Placebo	22
Barst et al. (2011)	MC, DB	IPAH, APAH, HPAH, Drugs and toxins induced	16 weeks	Bosentan (20 or 40 mg) + Tadalafil (40 mg)	42	Bosentan (20 or 40 mg)	45
Gatzoulis et al. (2019)	MC, DB	APAH	16 weeks	Macitentan (10 mg)	111	placebo	112
Rosenkranz et al. (2015)	MC, DB	APAH	12 weeks	Riociguat (2.5 mg)	13	placebo	11
Humbert et al. (2017)	MC, DB	APAH	12 weeks	Riociguat (2.5 mg)	71	placebo	25
Ghofrani et al. (2013)	MC, DB	IPAH, APAH, HPAH	12 Weeks	Riociguat (2.5 mg)	254	placebo	126

APAH: Connective tissue disease, Human immunodeficiency virus (HIV) infection, Portal hypertension, Congenital heart disease, and Schistosomiasis; DB: double-blind; HPAH: heritable pulmonary arterial hypertension; IPAH: idiopathic pulmonary arterial hypertension; MC: many centers; NR: not reported; SC: single-center.

**Table 2.** Patient characteristics of subjects included in the selected randomized controlled trials.

First author (year)	Age, years, mean (SD)		Sex, %				Cause of PAH, *n*				Baseline 6MWD, m Mean (SD)		WHO/NYHA functional class, *n*							
	Tre	Com	Tre		Com		Tre		Com		Tre	Com	Tre				Com			
			Male	Female	Male	Female	IPAH	Other	IPAH	Other			I	II	III	IV	I	II	III	IV
McLaughlin et al. (2010)	55	52	19.3	80.7	18.3	81.7	115		120		346 (63)	351 (69)	0	0	112	3	0	0	118	2
White et al. (2019)	45,6 (15.7)	44.8 (15.6)	20.5	79,5	78,2	21.8	219	127	216	128	392.9 (92.5)	398.5 (100.0)	9	205	131	1	13	228	103	0
Jing et al. (2013)	40.6	42.5	26	74	22	78	233		116		332.3 (71.6)	325.2 (77.1)	0	83	142	0	0	42	70	0
Hiremath et al. (2010)	30	36	37	63	43	57	30		14		–	–	0	0	29	1	0	0	13	1
Simonneau et al. (2012)	54.8 (16.8)	53.8 (16.3)	18.2	81.8	20	80	24	9	7	3	396.2 (71.4)	350.3 (123.5)	0	15	18	0	0	2	8	0
Coghlan et al. (2018)	50.6 (15.0)	50.7 (14.2)	20.1	79.9	20.8	79.2	106	73	118	79	359.7 (80.97)	358.7 (79.73)	0	55	122	0	0	60	133	0
Sitbon et al. (2015)	48.2 (15.19)	47.9 (15.55)	20.4	79.6	19,9	80.1	312	262	337	129	358.5 (76.31)	348.0 (83.23)	4	274	293	3	5	255	314	8
Galie et al. (2009)	53 (16)	55 (15)	24	76	21	79	50	32	54	28	338 (74)	343 (84)	0	28	54	0	1	23	56	2
Bermejo et al. (2018)	70	73	27	73	19	81	104		96		361	342	8	51	42	0	8	44	43	0
Galiè et al. (2015)	58 (11)	61 (10)	33	67	33	67	5	7	4	2	–	–	1	6	4	1	0	4	2	0
Rubin et al. (2015)	50 (16)	49 (16)	19	81	20	80	136	95	74	35	364 (67)	378 (66)	2	42	55	0	3	43	54	2
Galiè et al. (2008)	53 (14)	48 (16)	16	84	12	88	42	25	43	24	340 (77)	342 (73)	1	20	40	6	2	23	41	1
Galiè et al. (2008)	50 (16)	51 (14)	19	81	32	68	41	22	42	23	355 (84)	347 (84)	1	28	33	1	0	34	29	1
White et al. (2019)	50 (16)	53 (15)^a^	34	66	20	80	253		247		–	–	0	76	177	0	0	79	168	0
	50 (16)	52 (15) ^b^	34	66	18	82	253		126		–	–	0	76	177	0	0	38	88	0
	50 (16)	54 (15)^c^	34	66	22	78	253		121		–	–	0	76	177	0	0	41	80	0
Kuwana et al. (2020)	59.1 (12.1)	58.3 (13.0)	14	86	12	88	0	117	0	99	326 (89)	330 (96)	0	33	84	0	0	30	69	0
Baughman et al. (2014)	55 (8.8)	55 (10.1)	22	78	25	75	0	23	0	12	343 (95.5)	303 (78.8)	0	10	13	0	0	6	6	0
Rubin et al. (2002)	48.7 (15.8)	47.2 (16.2)	21	79	22	78	0	144	0	69	330 (74)	344 (76)	0	0	130	14	0	0	65	4
Galiè et al. (2006)	37.2 (12.0)	44.2 (8.5)	38	62	41	59	0	37	0	17	331.9 (82.8)	366.4 (67.5)	–	–	–	–	–	–	–	–
Badesch et al. (2002)	52.2 (12.2)	47.4 (14.0)	19	81	0	100	17	4	10	1	360 (86)	355 (82)	0	21	0	0	0	11	0	0
Galiè et al. (2008)	45.2 (17.9)	44.2 (16.5)	24	76	37	63	54	39	58	34	438 (86)	431 (91)	–	–	–	–	–	–	–	–
Ni (2018)	46.8 (8.24)	47.1 (7.05)	62	38	69	31	0	26	0	26	318.6 (90.4)	320.1 (92.4)	–	–	–	–	–	–	–	–
McLaughlin et al. (2015)	52.9 (15.4)	54.7 (15.7)	21.4	78.6	26.9	73.1	99	60	114	61	363.1 (78.5)	357.6 (73.1)	0	71	88	0	0	69	104	2
Iversen et al. (2010)	55.2 (15.1)	56.9 (14.1)	26	74	23	77	53		50		354.4 (73.1)	350.4 (87.6)	0	20	29	1	0	15	38	0
Vizza et al. (2017)	26.2 (6.4)	26.6 (6.7)	52	48	57	43	0	31	0	30	–	–	0	17	14	0	0	18	12	0
Pulido et al. (2013)	44.5 (16.2)	46.7 (17.0)	24.6	75.4	26.1	73.9	278	214	124	126	364 (95.5)	352 (110.6)	1	258	221	10	0	129	116	4
Sitbon et al. (2019)	58.0 (8.7)	59.0 (9.5)	51	49	48	52	0	43	0	41	385.8 (100)	383.2 (108.9)	1	27	15	0	1	23	18	0
Sitbon et al. (2019)	45 (15)	46 (17)	24	76	27	73	91	63	80	74	364 (97)	360 (111)	0	73	77	4	0	78	74	2
Jansa and Pulido (2018)	52 (12)	51 (14)	23.3	76.7	18.7	81.3	60		64		356 (87)	343 (71)	0	36	21	3	0	35	27	2
De-Zhen and An-Meng (2020)	11.5 (2.17)	11.5 (2.19)	56	44	53	47	0	43	0	43	–	–	–	–	–	–	–	–	–	–
Jian-zhou et al. (2018)	45.8 (16.3)	45.1 (14.4)	35.4	64.6	41.5	58.5	35	30	28	37	362 (94)	383 (93)	0	31	34	0	0	33	32	0
McLaughlin et al. (2006)	51 (14)	49 (15)	21	79	21	79	17	17	20	13	331 (64)	340 (73)	0	0	35	1	0	1	30	2
Hoeper et al. (2006)	48 (14)	56 (13)	24	76	21	79	21	0	19	0	317 (74)	296 (79)	–	–	–	–	–	–	–	–
Galiè et al. (2015)	54.5 (14.3)	54.2 (14.9)^d^	26	74	19	81	127	126	138	109	353.5 (87.9)	351.7 (91.8)	0	76	177	0	0	79	168	0
	54.5 (14.3)	53.9 (14.7)^e^	26	74	21	79	127	126	54	72	353.5 (87.9)	354.2 (92.3)	0	76	177	0	0	38	88	0
	54.5 (14.3)	54.5 (15.2)^f^	26	74	17	83	127	126	66	55	353.5 (87.9)	349.2 (91.6)	0	76	177	0	0	41	80	0
Badesch (2000)	53.0 (13.1)	57.3 (10.3)	9	91	18	82	0	56	0	55	–	–	0	1	42	13	0	4	45	6
Barst et al. (1996)	40 (3)	40 (2)	24	76	30	70	0	41	0	40	316 (18)	272 (23)	0	0	31	10	0	0	29	11
Barst (1997)	37	35	36	64	25	75	11	0	12	0	246	205	0	1	9	1	0	1	6	5
Galiè et al. (2002)	51 (15)	49 (17)	19	81	30	70	43	24	42	28	345 (77)	344 (79)	0	23	44	0	1	32	34	3
Wilkins et al. (2005)	41.1	44.4	17	83	21	79	11	1	12	2	304.6 (74.1)	290 (88.5)	–	–	–	–	–	–	–	–
Ronald (2004)	54 (2)	48 (2)	7	93	12	88	0	41	0	49	280 (13)	296 (13)	0	3	29	9	0	6	38	5
Tapson et al. (2012)	51	50	15	85	20	80	174		176		346.1 (71.4)	345.4 (75.5)	2	41	127	4	1	31	139	5
Simonneau et al. (2014)	47.8 (12.9)	47.5 (13.2)	18	82	23	77	107	27	105	28	348.9 (71.4)	341.6 (77.3)	1	34	88	10	2	34	87	6
Barst et al. (2003)	42 (2)	42 (2)	13	87	16	84	0	60	0	56	433 (11)	445 (10)	0	33	27	0	0	28	28	0
Humbert et al. (2004)	45 (17)	47 (19)	45	55	23	77	17	5	10	1	–	–	0	0	17	5	0	0	8	3
Jian-Yong et al. (2018)	41 (8)	42 (11)	14%	86%	15%	85%	0	35	0	41	212.9 (55.1)	219.7 (35.2)	–	–	–	–	–	–	–	–
Tapson et al. (2013)	51.5	50.4	24	76	20	80	157		153		329.4 (69.2)	336.8 (63.5)	0	43	110	3	0	37	115	0
Zhuang et al. (2014)	52 (12)	51 (14)	23.3	76.7	18.8	81.3	60		64		356 (87)	343 (71)	0	36	21	3	0	35	27	2
Jing et al. (2011)	32 (12)	29 (8)	18	82	15	85	25	19	14	6	395 (80)	388 (83)	0	21	23	0	0	9	11	0
Barst et al. (2011)	50.0 (12.9)	51.7 (16.1)	21	79	22	78	42		45		360.9 (75.3)	348.5 (84.9)	1	17	24	0	0	13	31	1
Gatzoulis et al. (2019)	33	31	28.1	71.9	39.3	60.7	0	114	0	112	368.7 (74.5)	380.3 (76.3)	0	69	45	0	0	66	46	0
Rosenkranz et al. (2015)	35 (14)	40 (16)	13	87	17	83	0	15	0	12	369 (78)	360 (59)	0	10	5	0	0	7	5	0
Ghofrani et al. (2013)	51 (17)	51 (17)	20	80	22	78	149	105	42	84	361 (68)	368 (75)	5	108	140	1	4	60	58	3

Com: comparator; IPAH: idiopathic pulmonary arterial hypertension; Other: connective tissue disease, human immunodeficiency virus (HIV) infection, portal hypertension, congenital heart disease, schistosomiasis and heritable pulmonary arterial Hypertension; 6MWD: 6-min walking distance; Tre: treatment.

^a^The comparator was placebo; ^b^the comparator was ambrisentan; ^c^the comparator was tadalafil; ^d^the comparator was placebo; ^e^the comparator was ambrisentan; ^f^the comparator was tadalafil.

### Statistical analysis

**Figure 3.**
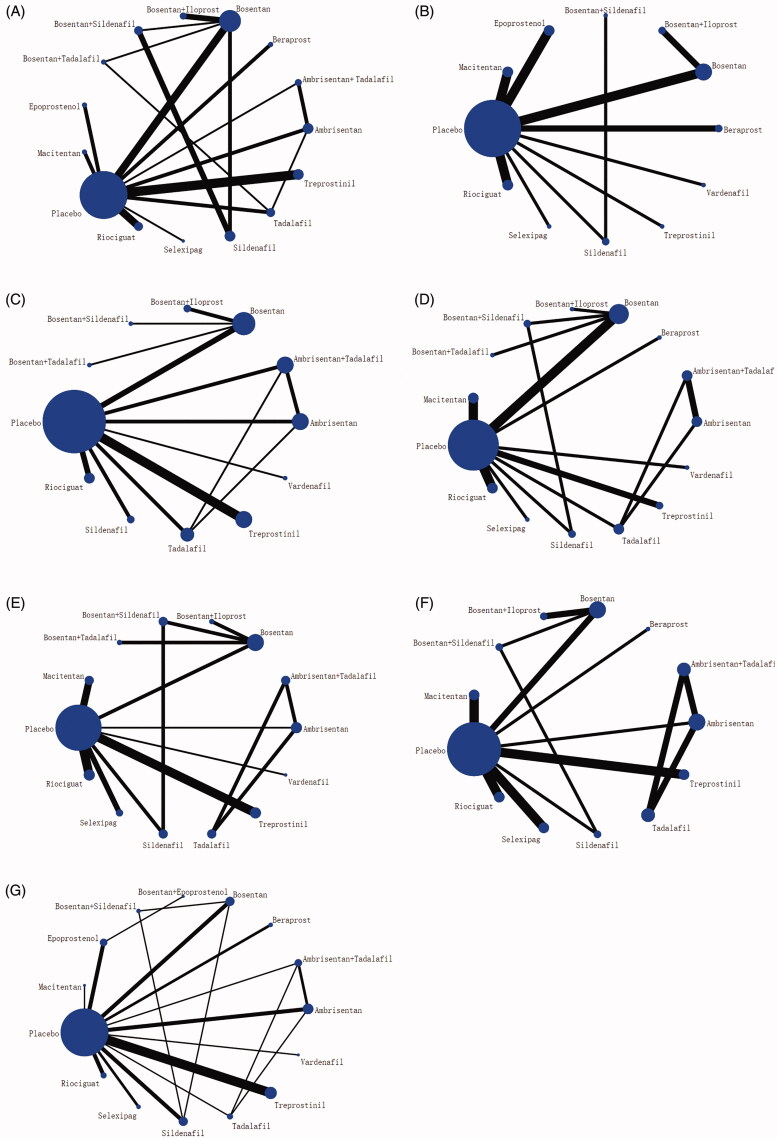
Network diagrams of comparisons on seven outcomes of different treatments in patients with pulmonary arterial hypertension. (A) 6-Minunt Walking Distance(6MWD) Change. (B) Mean Pulmonary Arterial Pressure (mPAP) Change. (C) Clinical worsening. (D) WHO Functional Class (FC) Improvement. (E) Adverse events (AEs). (F) Serious adverse events (SAEs). (G) All-cause mortality. The network plots show how a comparison of different treatments. Each vertex represents a type of treatment vertexes’ size represents the intervention sample size. The thickness of the straight line represents the number of trials compared.

The node-splitting method is used for inconsistency testing to analyze all outcome indicators. The results indicated that all direct and indirect evidence was consistent, suggesting that meta-analysis should be performed using a concordant model (*P* > 0.05). The node-splitting method-specific results are depicted in Supplementary Table 2. The network of comparisons for efficacy and safety were shown in Figure 3.

## The primary efficacy outcome: 6MWD

In terms of 6MWD, a total of 40 studies were included. The results showed that Bosentan combined with Sildenafil (WMD, 98.53, 95% CI, 69.13–127.94), Sildenafil (WMD, 79.24, 95% CI, 49.05–109.42), Bosentan combined with Iloprost (WMD, 69.16, 95% CI, 46.39–91.93), Bosentan combined with Tadalafil (WMD, 67.48, 95% CI, 44.41–90.55), Bosentan (WMD, 55.68, 95% CI, 43.46–67.90), Epoprostenol (WMD, 46.94, 95% CI, 32.36–61.52), Beraprost (WMD, 34.04, 95% CI, 9.18–58.91), Ambrisentan combined with Tadalafil (WMD, 24.86, 95% CI, 10.63–39.09), Riociguat (WMD, 23.94, 95% CI, 10.06–37.83), Macitentan (WMD, 17.10, 95% CI, 1.09–33.11) and Troprostacyclin (WMD, 14.77, 95% CI, 6.91–22.63) were statistically superior to placebo. According to the SUCRA, Bosentan combined with Sildenafil (99.0%), Sildenafil (87.5%), Bosentan combined with Iloprost (82.3%), and Bosentan combined with Tadalafil (80.9%) have the highest probabilities of being the best treatment options. The results of subgroup analysis showed that the mode of administration had no significant effect on the 6MWD. WMD for 6MWD from network meta-analysis was shown in Supplementary Table 3. SUCRA results were demonstrated in Table 3. The results of subgroup analysis were shown in Supplementary Table 4.

**Table 3.** Surface under the cumulative ranking curve (SUCRA) results for outcomes.

Treatment	6MWD change	mPAP change	WHO FC improvement	Clinical Worsening	death	AEs	SAEs
Bosentan + Sildenafil	0.990	0.844	0.464	0.484	0.570	0.844	0.444
Sildenafil	0.875	0.321	0.601	0.536	0.419	0.773	0.117
Bosentan + Iloprost	0.823	0.959	0.948	0.850	–	0.155	0.567
Bosentan + Tadalafil	0.809	–	0.599	0.855	–	0.445	–
Bosentan	0.700	0.613	0.457	0.692	0.641	0.751	0.251
Epoprostenol	0.631	0.453	–	–	0.644	–	–
Beraprost	0.513	0.230	0.412	–	0.550	–	0.629
Ambrisentan + Tadalafil	0.426	–	0.757	0.563	0.632	0.043	0.587
Riociguat	0.409	0.435	0.328	0.216	0.522	0.596	0.589
Selexipag	0.397	0.638	0.387	–	0.225	0.669	0.609
Macitentan	0.303	0.609	0.246	–	0.252	0.807	0.336
Treprostinil	0.270	0.274	0.242	0.221	0.516	0.807	0.473
Tadalafil	0.177	–	–	0.513	0.420	0.259	0.750
Ambrisentan	0.142	–	0.604	0.225	0.787	0.196	0.789
Placebo	0.038	0.074	0.070	0.038	0.191	0.769	0.360
Vardenafil	–	0.549	0.749	0.806	0.872	0.364	–
Bosentan + Epoprostenol	–	–	–	–	0.253	–	–

The surface under the cumulative ranking curve (SUCRA) was used to calculate each intervention’s probability. The larger the SUCRA value, the better the rank of the intervention.

AE: adverse event; 6MWD: 6-minute walking distance; mPAP: mean pulmonary arterial pressure; WHO FC improvement: WHO functional class (FC) improvement; SAE: serious adverse events.

## The secondary efficacy outcomes: mPAP

In terms of mPAP, a total of 22 studies were included. The results showed that Bosentan combined with Iloprost (WMD, −13.89, 95% CI, −19.56–8.22), Bosentan combined with Sildenafil (WMD, −10.80, 95% CI, −19.07–2.53), Bosentan (WMD, −5.89, 95% CI, −9.84–1.9), Macitentan (WMD, −5.76, 95% CI, −9.34–2.19), Epoprostenol (WMD, −4.14, 95% CI, −7.99–0.29) and Riociguat (WMD, −3.91, 95% CI, −7.65–0.17) were superior to placebo for lowering mPAP. According to SUCRA, Bosentan combined with Iloprost ranked first among all the treatments (SUCRA of 95.9%), followed by Bosentan combined with Sildenafil (SUCRA of 84.4%). WMD for mPAP from network meta-analysis was shown in Supplementary Table 5. SUCRA results were demonstrated in Table 3.

## WHO FC improvement

As far as the WHO FC improvement, there were 21 studies included in total. The improvement was statistically significant for Bosentan combined with Iloprost (OR, 14.81, 95% CI, 2.83–77.58), Ambrisentan combined with Tadalafil (OR, 4.02, 95% CI, 1.26–12.82), Tadalafil (OR, 3.14, 95% CI, 1.05–9.42), Sildenafil (OR, 2.58, 95% CI, 1.18–5.66), Bosentan (OR, 2.00, 95% CI, 1.30–3.06), Riociguat (OR, 1.59, 95% CI, 1.07–2.36) versus Placebo. In the SUCRA, Bosentan combined with Iloprost ranked first among all the treatments (SUCRA: 95.4%), followed by Ambrisentan combined with Tadalafil (SUCRA: 79.8%). OR for WHO FC improvement from network meta-analysis was shown in Supplementary Table 6. SUCRA results were demonstrated in Table 3.

## Clinical worsening

In the case of Clinical Worsening, a sum of 24 studies were included. In network meta-analysis, the proportion of clinical worsening was significantly reduced in Bosentan combined with Tadalafil (OR, 0.08, 95% CI, 0.01–0.55), Bosentan combined with Iloprost (OR, 0.09, 95% CI, 0.02–0.48), Vardenafil (OR, 0.09, 95% CI, 0.01–0.92), Bosentan (OR, 0.21, 95% CI, 0.10–0.43), Ambrisentan combined with Tadalafil (OR, 0.30, 95% CI, 0.16–0.58), Sildenafil (OR, 0.31, 95% CI, 0.15–0.68), Tadalafil (OR, 0.34, 95% CI, 0.17–0.67) and Treprostinil (OR, 0.66, 95% CI, 0.45–0.97), versus Placebo. Based on the NMA model for reducing the occurrence of clinical worsening, targeted drugs for pulmonary hypertension (PH) can be ranked as follows: Bosentan combined with Tadalafil (SUCRA 85.5%) > Bosentan combined with Iloprost (SUCRA 85.0%). OR for Clinical Worsening from network meta-analysis were shown in Supplementary Table 7. SUCRA results were demonstrated in Table 3.

## All-cause death

For efficacy outcomes of all-cause death, 42 studies were included in total. The results showed that Ambrisentan (OR, 0.29, 95% CI, 0.11–0.78) and Treprostinil (OR, 0.58, 95% CI, 0.41–0.84) were statistically superior to placebo. According to the SUCRA, Vardenafil (87.2%), Ambrisentan (78.7%) have the highest probabilities of being the best therapeutic options. OR for all-cause death from network meta-analysis were shown in Supplementary Table 8. SUCRA results were demonstrated in Table 3.

## Safety outcomes

### AEs

As far as AEs, a total of 37 studies were included. Compared to Placebo, Ambrisentan combined with Tadalafil (OR, 14.15, 95% CI, 4.07–49.16), Ambrisentan (OR, 5.75, 95% CI, 2.57–12.88), Tadalafil (OR, 4.82, 95% CI, 1.42–16.36), Treprostinil (OR, 3.31, 95% CI, 1.53–7.19) significantly increased the incidence of AEs. According to the SUCRA, Bosentan combined with Sildenafil (84.4%), Macitentan (80.7%), and Sildenafil (77.3%) have the highest probabilities of being the best therapeutic options. OR for AEs from network meta-analysis were shown in Supplementary Table 9. SUCRA results were displayed in Table 3.

### SAEs

In the case of SAEs, a total of 26 studies were included. There was no statistically significant difference between targeted drugs and Placebo. According to the SUCRA, Ambrisentan (78.9%) and Tadalafil (75.0%) have the highest probabilities of being the best therapeutic options. ORs for SAEs from network meta-analysis were shown in Supplementary Table 10. SUCRA results were displayed in Table 3.

### Sensitivity analyses

The sensitivity analyses were consistent with the primary analysis results. The results were shown in Supplementary Table 11.

## Discussion

In this network meta-analysis, Bosentan combined with Sildenafil; Sildenafil, Bosentan combined with Iloprost; and Bosentan combined with Tadalafil significantly increased 6MWD. Bosentan combined with Iloprost and Bosentan combined with Sildenafil significantly reduced mPAP. Bosentan combined with Iloprost, and Ambrisentan combined with Tadalafil significantly improved WHO FC. Bosentan combined with Tadalafil and Bosentan combined with Iloprost significantly reduced clinical worsening. Ambrisentan and Ambrisentan combined with Tadalafil significantly increased the incidence of AEs. No treatment regimen has been shown to reduce the incidence of SAEs significantly. Ambrisentan has demonstrated clear benefits in reducing all-cause mortality. Bosentan combined with Sildenafil; Bosentan combined with Tadalafil; Bosentan combined with Iloprost and Sildenafil have relatively good efficacy and safety.

In our study, we found that the combination therapy performed better than placebo in PAH patients at the end of this study, which is in line with previous reviews on the same matter. Still, they are considerably more precise because of our larger quantity of data and resulting statistical power.

At present, combination therapy is not limited to PAH and has been widely used in other chronic diseases such as heart failure (Burnett et al., 2017), hypertension (Paz et al., 2016), tumours (Bennouna and Moreno Vera, 2016) etc. The combination therapy of these patients has a better effect than the single-drug therapy, which may be related to additive or even synergistic effects of the combined treatment against multiple pathways in the pathogenesis of PH. There are at least two benefits of combination therapy: (1) make the treatment reaches the target as soon as possible; (2) reduce the therapeutic dose and minimize AEs. Combination therapies are divided into sequential combination therapy and initial combination therapy. Sequential combination therapy is the most widely used treatment strategy in clinical practice and clinical trials. For patients who have already adopted a treatment plan (using single-drug or even combination therapy), sequential combined treatment is required if they still do not reach a low-risk state. At present, several clinical trials have confirmed that sequential combination therapy can achieve better efficacy than single-drug therapy (McLaughlin et al., 2010; Ghofrani et al., 2013). A meta-analysis that included 4095 PAH patients showed that compared with single-drug therapy, sequential combination therapy reduced the risk of clinical deterioration by 35% (Lajoie et al., 2016). Besides, for this type of treatment, the incidence of additive side effects is lower than the initial combination therapy because the patient must first adapt to the systemic vasodilation effect of one drug before starting another drug.

Consequently, adding therapy to the existing treatment generally results in the same side effect as the initial treatment (Burks et al., 2018). It needs to be managed and monitored when the new therapy is introduced. For the initial combination therapy, it was confirmed for the first time that the WHO FC II–III PAH patients received combination therapy more benefits in the Research of AMBITION in 2015 (Galiè et al., 2015). Since then, more and more evidence, including large RCTs, supports initial combination therapy as an effective treatment strategy for PAH (Hassoun et al., 2015; Han et al., 2017; D’Alto et al., 2018). Therefore, the 2018 WORLD SYMPOSIUM ON PULMONARY HYPERTENSION (WSPH) emphasized the importance of combination therapy for PAH patients and recommended low/intermediate/high-risk PAH patients with negative acute vascular reactivity test should first consider the combination of ERA and PDE5i (Qin and Zhi-Hong, 2020). ESC/ERS explicitly recommends the initial combination therapy for high-risk PAH patients, and the treatment should include intravenous prostacyclin analogs. A recent retrospective study found that the triple upfront combination therapy with Ambrisentan, Tadalafil, and subcutaneous Treprostinil significantly improved the clinical and hemodynamics of patients with severe irreversible PAH and was related to right heart reverse remodeling (D’Alto et al., 2020). However, the potential adverse reactions should be considered when using the initial combination therapy for any disease. Although ERA, PDE5i, SGC, and ProsA have different action mechanisms, they can induce vasodilation (Ataya et al., 2016). Thus, compared with monotherapy, the initial combination therapy may cause more AEs.

In terms of outcomes, we found that ERA and PDE5i combined treatments, such as Bosentan combined with Sildenafil, Bosentan combined with Tadalafil, and ERA and ProsA combined treatments, such as Bosentan combined with Iloprost, have a higher probability of more improve 6MWD and cardiac function. Although patients were prone to edema, headache, diarrhea, dizziness, and other adverse reactions (Galiè et al., 2008), most patients could tolerate them. Consequently, we considered that ERA combined with PDE5i or ERA combined with ProsA of PAH in the early stage could prevent irreversible remodeling of pulmonary vessels (O'Connell et al., 2013), to get more significant benefits. However, continuous monitoring of blood concentration of patients is needed to judge the progress of PAH (Coghlan et al., 2018) in patients and timely respond to possible AEs when combined treatment regimens are used. Besides, the interaction between drugs should also be considered. For example, the pharmacokinetic interaction between Bosentan and Sildenafil may cause insufficient sildenafil drug plasma concentrations (Grünig et al., 2017). Therefore, the routine monitoring of Sildenafil-Bosentan plasma concentrations is necessary. If patients with inadequate treatment response, the switch from Bosentan to other alternative ERA (Ambrisentan or Macitentan) should be considered (Apitz and Schranz, 2018). However, expect for Sildenafil + Bosentan, Sildenafil + Tadalafil, Ambrisentan + Tadalafil, different ERA + PDE5i still no RCTs to confirm the efficacy and safety. It is necessary to be cautious in clinical treatment. For patients receiving long-term single-agent therapy (>5–10 years) with stable and low-risk symptoms, Age >75, Suspected pulmonary veno-occlusive disease/pulmonary capillary hemangiomatosis (PVOD/PCH), and other patients not recommended to use combined therapy. Although Sildenafil may cause headache, epistaxis, and muscle pain, the benefit of Sildenafil may be more significant. It is crucial to watch the dosage during treatment (Spradley, 2012).

## Limitations

Although the results of our network meta-analysis are relatively comprehensive, some limitations may affect the accuracy of each result. Firstly, the differences in patient population, baseline clinical value, drug dose and duration of treatment among all RCTs may affect the results. Besides, most of the comparisons about combined treatments are only indirect and affected by other covariates. Therefore, these results should be interpreted with caution in the absence of a direct comparison of combination therapies.

## Conclusion

We recommend PDE5i + ERA and ProsA + ERA as the best choice for clinical treatment of PAH patients. But in the future, clinicians should choose according to the patient’s individualized situation and the patient’s requirements when developing cure strategies. We hope that these results will assist in shared decision making between patients, and their clinicians.

## Data Availability

The datasets used and analyzed during the current study are available from the corresponding author on reasonable request.

## Modification of the protocol

If we need to amend this protocol, we will give the date of each amendment, describe the change and give the rationale in this section. Changes will not be incorporated into the protocol.
